# Messrs. Ticknor & Fields

**Published:** 1864-11

**Authors:** 


Messrs. Ticknor & Fields, Boston, will shortly begin the publication of a new juvenile magazine, entitled *Our Young Folks:* an Illustrated Monthly Magazine for Boys and Girls ; edited by J. T. Trowbridge, Gail Hamilton, Lucy Larcom. The staff of contributors will include many of the most popular writers of Juvenile Works in America and in England. Terms, $2.00 per year.

Among the interesting specimens collected in the Medical department at Bowdoin College, says the Lewistown Journal, is the ossified body of an infant child, 27 years old, which had never been born, presented to the College last spring by the late Dr. Prescott, of Farmington. Dr. P. was called to its mother twenty-seven years ago, but was obliged to leave it unborn, in which condition it remained until last spring, when the mother died, and he made a post mortem examination, and withdrew the fœtus, now an ossified body weighing about six pounds. Within that period of twenty-seven years, that lady had become the mother of three children who are now living.

